# The role of cocoa flavanols in modulating peripheral and cerebral microvascular function in healthy individuals and populations at-risk of cardiovascular disease: a systematic review

**DOI:** 10.1186/s12937-025-01121-9

**Published:** 2025-04-11

**Authors:** Sophie Richardson, Janice Marshall, Catarina Rendeiro

**Affiliations:** 1 School of Biomedical Sciences, College of Medical and Dental Sciences, University of Birmingham, Edgbaston, Birmingham, B15 2TT UK; 2 School of Sport, Exercise and Rehabilitation Sciences, University of Birmingham, Birmingham, B15 2TT UK; 3 Centre for Human Brain Health, University of Birmingham, Birmingham, UK

**Keywords:** Microvasculature, Cocoa, Flavanols, Flavonoids, Vasodilation

## Abstract

**Background:**

Cocoa flavanols (CFs) are polyphenolic molecules with proposed cardioprotective effects. Whilst extensive evidence supports their ability to ameliorate vasodilator responses within conduit vessels, their actions in the microvasculature are less clear. This systematic review of the literature aimed to determine whether CF interventions lead to improvements in microvascular vasodilator responses in healthy populations and those with increased cardiovascular disease risk.

**Methods:**

Database searches were conducted up to September 2023 using Medline, Embase, Pubmed and Web of Science Core Collection to identify randomised, placebo-controlled, human studies investigating the effect of CF interventions on the microvasculature (at rest and vasodilator responses). All studies were assessed for risk of-bias according to Cochrane Collaboration recommendations for randomised-controlled trials, data were extracted from studies and findings collated by vote-counting.

**Results:**

Searches identified 511 unique articles for screening, of which 19 were selected for data extraction. Vasodilator responses were enhanced in 85.7% (80.4–91.0%, *p* = 0.013) of all acute studies (*n* = 13), and in 81.8% (74.1–89.4%, *p* = 0.065) of studies in healthy subgroups (*n* = 11). Importantly, this effect was apparent in all studies with ‘low risk of bias’ (n = 8, p = 0.008). In contrast, there was no effect of acute CF interventions at rest. For chronic studies (n = 7), the effect of CFs was less clear, with a significant benefit reported at rest only, in all young, healthy subgroups (n = 7, p = 0.016), but no evidence of improvements in vasodilator responses.

**Conclusions:**

CFs have the potential to improve microvascular function, particularly in healthy individuals, with benefits appearing more pronounced following acute CF supplementation. Despite this, interpretations are limited by the small number of comparable studies identified and the heterogeneity of populations studied. Overall, the effects of CFs on the microvasculature seem to be less consistent than previous evidence in the macrovasculature, suggesting that the microvessels may be less susceptible to the effect of CFs than conduit arteries.

**Registration:**

The PROSPERO registration number for this review is CRD42023483814.

**Supplementary Information:**

The online version contains supplementary material available at 10.1186/s12937-025-01121-9.

## Background

Diets rich in flavonoids, polyphenolic molecules derived from plants, have been associated with reductions in cardiovascular disease (CVD) risk [1, 2]. In particular, flavanols, a subgroup of flavonoids that can be found in grapes, apples, tea and cocoa, have well-established cardioprotective effects [3]. For example, early epidemiological studies suggest significant reductions in blood pressure and cardiovascular mortality in populations with higher levels of cocoa intake [4, 5]. This has been further supported by randomised controlled trials (RCTs) showing that cocoa flavanol (CF) supplementation reduces systolic and diastolic blood pressure in interventions ranging from 2 to 18 weeks duration, with greater benefits being observed in individuals with hypertension in comparison to normotensives [6, 7].

In addition to benefits in blood pressure, there is also extensive evidence that CFs can improve endothelium-dependent dilatation (EDD), as measured by flow-mediated dilatation (FMD) of the brachial artery, which has predictive value for future risk of CVD [8]. Human studies demonstrate improvements in FMD following both acute (1–3 h) and chronic (2–18 weeks) ingestion of CFs in healthy young [9–11] and elderly [10, 12] populations, as well as in individuals with increased CVD risk, such as smokers [13, 14], those who are overweight or obese [15–17], and individuals with hypertension [18], type-2 diabetes [19] and peripheral artery disease [20]. The benefits of CFs on brachial artery endothelial function have been reviewed extensively [21, 22] and a non-linear dose-response relationship has been observed in chronic studies, with maximal FMD improvements obtained at 500–700 mg cocoa flavanols and ~ 95 mg (-)-epicatechin [23–25].

By contrast, studies investigating the effect of CFs on the microvasculature are more limited. Venous occlusion plethysmography (VOP), Laser Doppler and near-infrared spectroscopy (NIRS) are typically used in skeletal muscle, cutaneous and cerebral vascular beds to non-invasively monitor changes in blood flow induced by responses in arterial resistance vessels and microvessels [26]. These techniques can be used to measure resting vascular function and EDD, such as reactive hyperaemia (RH) and responses to local heating, exercise, or mental stress and together they provide a robust way of assessing endothelial function across different microvascular beds.

Similarly to the macrovasculature, changes in EDD in the microvasculature can also be predictive of CVD progression [27]. Indeed, some studies suggest that peak hyperaemic flow in smaller vessels following release of arterial occlusion (i.e. peak RH), measured for example by VOP, provides a more robust prediction of cardiovascular health than brachial FMD [28, 29]. Microvascular dysfunction, which is evident in CVD, ageing and other associated risk factors [30–33], is also closely linked with impaired endothelial function and leads to structural and functional changes in the microvasculature [34, 35]. Importantly, functional declines within the microvasculature often precede macrovascular complications and can lead to development of pathological interactions and disease in both small and large vessels, across multiple organs [36–38]. Hence changes in endothelial function within the microvasculature may be useful in the early prediction of the progression of CVD. On this basis, it is important to establish the effect of CFs within the microvasculature and assess how this may differ from the benefits previously demonstrated in conduit vessels, as well as how potential benefits may vary between healthy individuals and those at increased risk of CVD.

Thus, the aim of the present study was to conduct a systematic review of the literature assessing the impact of CF supplementation on the microvasculature in order to establish whether CF interventions lead to improvements in microvascular vasodilator responses in healthy populations and those at risk of CVD.

## Methods

This systematic review was conducted following the Preferred Reporting System for Systematic Reviews and Meta-Analyses (PRISMA) guidelines (Additional File 1) [39]. The review has been registered with PROSPERO (registration number: CRD42023483814); this includes full details on sample search query, criteria for inclusion and exclusion, data extraction, and analysis as recommended according to the PRISMA-P guide [39, 40].

### Search strategy

Search terms were selected based on the PICO format [39, 41] and are detailed in Table 1; words within each column were listed with ‘OR’ and the ‘AND’ function was used to combine columns into a search list. As recommended by this model, terms were chosen according to Population (healthy adults, and those with increased CVD risk), Intervention (CF supplementation, in the form of dark chocolate, cocoa drink or pure CFs), Comparison (studies with a control group), and Outcome (reporting microvascular function). Systematic searches were conducted up to September 2023, using Medline (Ovid, 1946 onwards), Embase (Ovid, 1980 onwards), PubMed (1996 onwards), Web of Science Core Collection (Clarivate Analytics, 1900 onwards) and Cochrane Central Register of Controlled Trials (Wiley Interface, current issues).

**Table 1:** Systematic review search terms organised by the PICO format

Population	Intervention	Control	Outcome
Human Subjects Volunteers Patients Males Females Overweight Obes* Diabet* Hypertens* Elder* Young Menopaus*	Chocolate Cocoa Cacao	Placebo Control	Microcirc* Microvasc* Microvessels Capillar* Arteriol* Venul* Iontophoresis Blood flow Perfusion Vasodila* Laser doppler Video capillaroscopy Laser speckle NIRS Plethysmography

*Is truncation symbol used to include various word endings and spellings

### Selection criteria and screening

Screening was conducted using the Rayyan online screening tool [42]. Full citations were collated and screened initially by title and abstract to determine those for which the full text should be accessed. This was conducted separately by two independent reviewers, and any conflicts were settled by discussion with a third reviewer, as recommended by the Cochrane guidelines [43]. Reviews and meta-analyses identified during literature searches were manually evaluated for any additional studies not returned by the initial database searches. During full-text screening, studies were included according to the following criteria; (i) randomised studies with placebo control arm, or cross-over studies with sufficient washout time and appropriate blinding, (ii) CFs administered orally including in dark chocolate, or as pure individual flavanols, (iii) healthy, young adults or clinical populations with elevated CVD risk, (iv) studies reporting relevant outcome measures of microvascular function, such as cerebrovascular or peripheral blood flow (as measured by for example by Laser Doppler, NIRS or VOP), with sufficient data for comparison of control and treatment groups. Exclusion criteria included: (i) non-human studies, (ii) articles that did not report the dose of cocoa intervention, (iii) studies with no placebo group, (iv) studies investigating the effects of cocoa on other measures, such as macrovascular responses, or where the contribution of the microcirculation to the measure was unclear.

### Data extraction and synthesis

Data extraction was conducted by a single reviewer using a pre-prepared table including the following information; study characteristics (author, year of publication, journal), study design (including details on randomisation, placebo), population details (sample size, sex, age, health status), type of intervention (cocoa drink, capsule, dark chocolate), dosage and duration of intervention, and all available data on pre- and post- intervention for CFs and placebo. Where necessary, vascular measures were extracted from graphs if not given in the text. In studies where multiple doses of a single supplement, or multiple interventions or outcome measures were compared, all the available measures were initially extracted and then the most relevant selected to report so as to ensure consistency between studies wherever possible. For example, only the 1–2 h time-points were reported for acute studies as this is when blood CF concentrations are at their peak [44–46]. For NIRS studies, data are shown as oxyHb, a measure of the change in oxygenated haemoglobin or oxygen saturation (SO_2_), a measure of tissue oxygenation, as these were the most widely reported measures and provide insight into microvascular vasodilator behaviour [47, 48].

Tables of findings are arranged according to vascular bed studied and ordered by risk of bias. Study outcomes are presented for placebo and CFs from each study; these are shown as pre- and post- intervention or change from pre- intervention wherever possible. Each study is represented throughout by a letter and subgroups within a study are allocated numbers (i.e. for Heiss et al. (b_1_) represents the young group, and (b_2_) the elderly group). We had aimed to conduct a meta-analysis, however this requires at least two studies reporting the same outcome measures [49], and was not possible due to the heterogeneity of techniques, vasodilator stimuli and statistical methods across studies without enough information being provided to reduce these to the same format [50]. Instead, we synthesised findings from studies by ‘vote counting’, as recommended in the Cochrane Handbook for reviews where the limited availability of data does not even allow for summarising effect estimates, or combining p values, as preferable alternatives to meta-analysis [51]. This involves assigning each outcome a ‘vote’ depending on the direction (regardless of statistical significance) of the percentage change calculated between placebo and CFs; ‘1’ for a positive percentage change (i.e. higher with CFs) or ‘0’ for a negative change (i.e. higher with placebo) [52]. The assigned votes are shown in results tables, and these were used to determine groupings used in the Harvest plot, which presents the direction of effect and risk of bias for each outcome.

### Quality assessment

Cochrane Collaboration recommendations were used when assessing the quality of studies: based on five criteria covering random sequence generation; (1) selection bias (allocation concealment), (2) performance bias (blinding of participants and researchers), (3) detection bias (blinding of outcome assessment), (4) attrition bias (incomplete outcome data), and (5) reporting bias (selective reporting) [53]. Studies were given a bias rating for each domain based on a set of signalling questions, these being combined according to Cochrane recommendations to give an overall assessment of bias for the study. When fewer than 95% of original participants were included in the analysis, studies were considered to have ‘missing data’. Studies were judged overall ‘low risk’ if they were ‘low risk’ across all domains, ‘some concerns’ if concerns were raised for at least one domain, or ‘high risk’ either if any domain was judged ‘high risk’ or if there were multiple domains with concerns deemed sufficient to lower confidence in result. Overall bias ratings were important for considering bias as a potential cause for heterogeneity within the results, as recommended by established guidelines [41].

### Statistical analysis

Due to the lack of studies reporting similar enough outcomes for synthesis of findings by meta-analysis, alternative tools were used for analysis of our results. From the ‘votes’ assigned to each outcome, we calculated the proportion of studies which showed a beneficial effect of CFs, and this was reported alongside a 95% confidence interval estimated by Wilson intervals method [54]. Binominal tests were also used to calculate the probability of the overall direction of effect being true (*p* < 0.05 considered significant). This was conducted in Microsoft Excel using the formula ‘=2*BINOM.DIST(x,y,z, TRUE), where x is the smaller of the number of effects favouring the intervention or control, y is the total number of effects, and z is the null value (true proportion of effects favouring intervention = 0.5). Sensitivity analysis was also conducted, in which only studies judged overall as ‘low risk of bias’ were included in the analysis.

## Results

### Search results

**Fig. 1:**
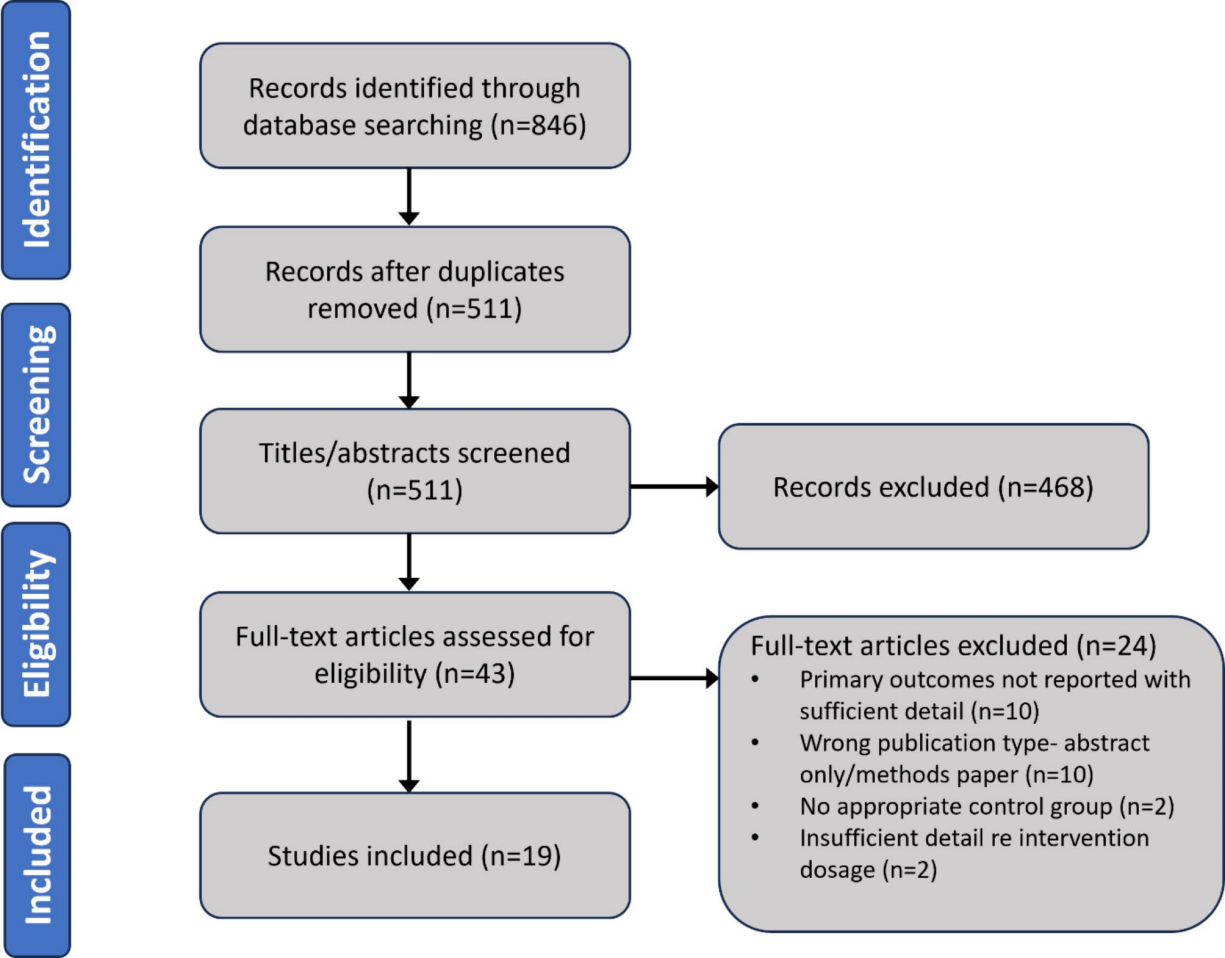
PRISMA flow diagram showing the stages from identification to selection of studies to include, as well as reasons for exclusion of full-text articles

The process of search, screening and selection of eligible studies is shown in Fig. 1. A total of 846 papers were identified through searches of Pubmed, Embase, Medline and Web of Science, of which 511 unique articles remained once duplicates had been removed. 468 records were removed during screening by title and abstract due to not meeting the inclusion criteria; for example, animal studies, studies using interventions other than CFs, or lack of clear microvascular outcome measures. Full texts for the remaining 43 articles were then assessed for eligibility, of which 24 were excluded due to the following reasons: incorrect publication type (abstract only or methods paper, *n* = 10); primary outcomes not reported, or without sufficient detail (*n* = 10), lack of detail regarding the intervention dosage (*n* = 2), or no suitable control group (*n* = 2).

### Risk of bias of included studies

**Fig. 2:**
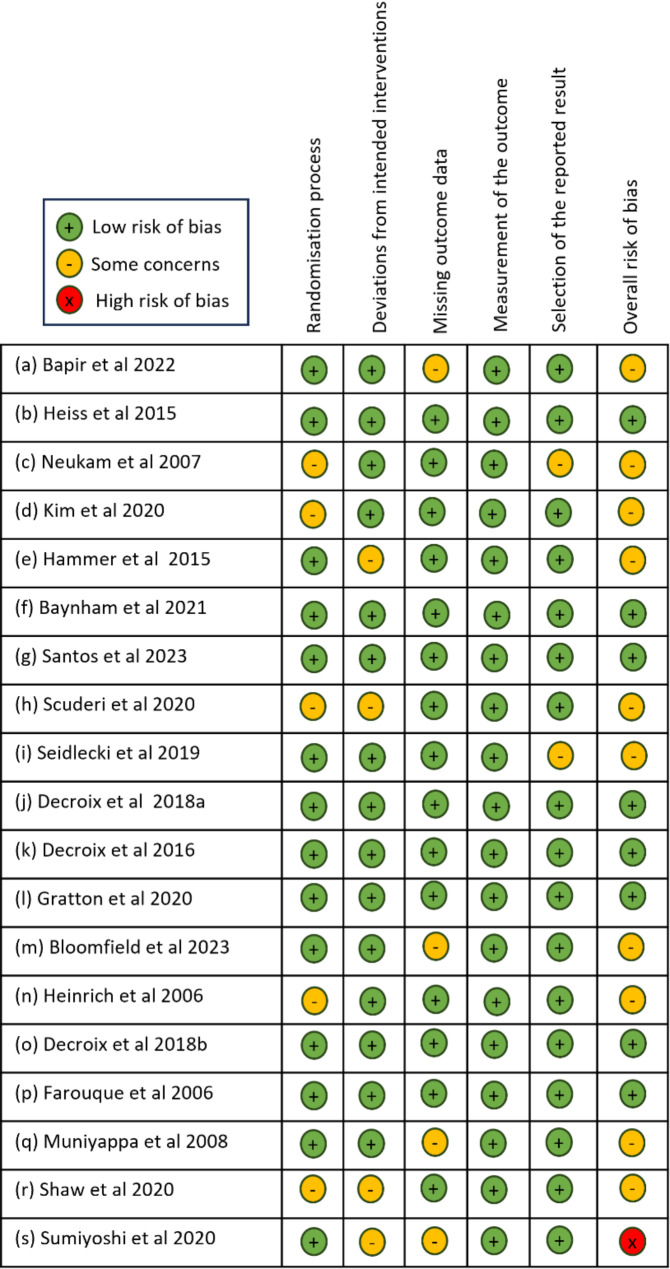
Risk of bias assessment according to the cochrane recommendations

Each study included was assessed for Risk of Bias using the Cochrane Risk of Bias assessment guidelines. The reviewer’s judgements for each domain, alongside the overall bias are shown for each study in Fig. 2. 8 of the included studies were considered ‘low risk of bias’, 10 were classified as ‘some concerns’ and only one study was deemed ‘high risk of bias’. Potential sources of concern for bias were largely due to missing data (n = 4), uncertainties surrounding the randomisation process (n = 5) or blinding issues, for example, use of white chocolate as placebo (n = 4).

### Study characteristics

Of the 19 studies that met the inclusion criteria, some used a single CF dose (*n* = 12) [45, 55–65], whilst others investigated effects of daily CF intervention over a longer period (*n* = 6) [66–71] and one study included both acute and chronic effects [72]. The characteristics of all included studies are displayed in Tables 2 and 3 for acute and chronic studies respectively; in each case they are arranged by vascular bed studied.

**Table 2:** Characteristics of included acute studies, including information on population studied and interventions used

	Study design (washout period)	Participants’ health	Number of participants	Age (yrs)	% female	Cocoa intervention type/dose	Control intervention type	Microcirculation studied
(a) Bapir 2022 [56]	Crossover	Healthy	*n* = 11	51 ± 10	72.7%	6 capsules- *Total flavs: 1350mg* *Epicatechin: 255mg*	6 capsules containing 3.9 g brown sugar- *Total flavs: 0mg*	Cutaneous (hands/feet)
		T2DM	*n* = 11	59 ± 10	27.3%			
(c) Neukam 2007 [45]	Crossover (14 days)	Healthy	*n* = 10	18–65	100%	Cocoa beverage (18 g cocoa powder in 100 ml water) *Total flavs: 329mg* *Epicatechin: 61.1mg*	Matched beverage (18 g cocoa powder in 100 ml water) *Total flavs: 27mg* *Epicatechin: 6.6mg*	Cutaneous
(d) Kim 2020 [62]	Crossover (min 7days)	(d_1_) Healthy WEs	*n* = 7	22 ± 4	42.9%	Cocoa beverage (in 250 ml water)- *Total flavs: 528mg* *Epicatechin: 47mg*	Matched placebo beverage (in 250 ml water) *Total flavs: 0mg*	Cutaneous (forearm)
		(d_2_) Healthy BAs	*n* = 7	22 ± 4	42.9%			
(e) Hammer 2015 [61]	Crossover (7 days)	PAD patients	*n* = 21	66.9 ± 7.41	28.6%	50 g dark choc *Total flavs: 780mg* *Epicatechin: 45mg*	50 g white choc *Total flavs: 0mg*	Cutaneous (forearm)
(b) Heiss 2015 [72]	Parallel	(b_1_) Healthy young (18-30yrs)	*n* = 22	26 ± 4.69	0%	Cocoa beverage (7 g cocoa powder in ~ 500 ml water) *Total flavs: 450mg* *Epicatechin: 64mg*	Cocoa beverage (7 g matched powder in ~ 500 ml water) *Total flavs: 0mg*	Cutaneous/ muscle (forearm)
		(b_2_) Healthy elderly (50-80yrs)	*n* = 20	60 ± 8.94	0%			
(f) Baynham 2021 [55]	Crossover (7 days)	Healthy (all White European)	*n* = 30	23 ± 4.3	0%	8.3 g cocoa powder (dissolved in 300 ml Buxton water) *Total flavs: 681.4mg* *Epicatechin: 150mg*	8.3 g matched cocoa powder (dissolved in 300 ml Buxton water) *Total flavs: 4.1mg* *Epicatechin: <4mg*	Skeletal muscle (forearm)
(g) Santos 2023 [63]	Crossover (1 month)	Healthy	*n* = 12	25 ± 4	53.8%	25 mg microencapsulated cocoa powder (dissolved in 250 ml water) *Total flavs: 80mg*	7 g microencapsulated Ovaltine *Total flavs: 9mg*	Skeletal muscle (forearm)
(h) Scuderi 2020 [64]	Crossover (72 h)	Healthy	*n* = 18 (36 eyes)	26.3 ± 1.5	44.4%	100 g dark chocolate *Epicatechin: 447mg*	100 g white chocolate *Total flavs: trace*	Retinal
(i) Siedlecki 2019 [65]	Crossover (7 days)	Healthy	*n* = 22	27.3 ± 11.1	59.1%	20 g dark chocolate *Total flavs: 400mg*	7.5 g milk chocolate *Total flavs: 5mg*	Retinal
(j) Decroix 2018a [59]	Crossover (7 days)	Healthy	*n* = 20	23.2 ± 4.3		4 capsules (1765 mg cocoa extract) *Total flavs: 530mg* *Epicatechin: 100mg*	4 matched capsules *Total flavs: 0mg*	Cerebral
(k) Decroix 2016 [58]	Crossover (7days)	Trained males	*n* = 12	30 ± 3	0%	Cocoa powder dissolved in 300 ml semi-skimmed milk *Total flavs: 903mg* *Epicatechin: 185mg*	Matched placebo powder dissolved in 300 ml semi-skimmed milk *Total flavs: 15mg* *Epicatechin 0mg*	Cerebral
(l) Gratton 2020 [60]	Crossover (2 weeks)	Healthy	*n* = 18	23.9 ± 7.3	0%	8.3 g cocoa powder (dissolved in 300 ml Buxton water) *Total flavs: 681.4mg* *Epicatechin: 150mg*	8.3 g matched cocoa powder (dissolved in 300 ml Buxton water) *Total flavs: 4.1mg* *Epicatechin: <4mg*	Cerebral
(m) Bloomfield 2023 [57]	Crossover (2 weeks)	Healthy	*n* = 12	26.1 ± 6.2	41.7%	Encapsulated cocoa powder (15 mg/kg body weight) *Mean total flavs: 1031mg* *Mean epicatechin: 145mg*	Encapsulated placebo cocoa powder *Mean total flavs: 0.82mg* *Mean epicatechin: n/a*	Cerebral

*Data shown are mean ± SD. T2DM = type 2 diabetes mellitus. PAD = peripheral artery disease. WE = White European*,* BA = Black African. Flavs = flavanols*

**Table 3:** Characteristics of included chronic studies, including information on population studied and interventions used

	Study design (washout period)	Participants’ health	Number of participants	Age (yrs)	% female	Study duration	Daily cocoa intervention type/dose	Daily control intervention type	Microcirculation studied
(n) Heinrich 2006 [68]	Parallel	Healthy females	*n* = 24 (12 per group)	18–65	100%	12 weeks (tested also at 6wks)	18 g powder in 100 ml water *Total flavs: 329mg* *Epicatechin: 61.1mg*	18 g powder in 100 ml water *Total flavs: 26.8 g* *Epicatechin: 1.6mg*	Cutaneous
(b) Heiss 2015 [72]	Parallel	(b_1_) Healthy young (18-30yrs)	*n* = 22	26 ± 4.69	0%	14 days	Cocoa beverage (7 g cocoa powder in ~ 500 ml water) *Total flavs: 450mg* *Epicatechin: 64mg*	Cocoa beverage (7 g matched powder in ~ 500 ml water) *Total flavs: 0mg*	Cutaneous/ skeletal muscle (forearm)
		(b_2_) Healthy elderly (50-80yrs)	*n* = 20	60 ± 8.94	0%				
(p) Farouque 2006 [67]	Parallel	CAD patients	CFs: *n* = 20 Control: *n* = 20	CFs:61 ± 9 Control: 61 ± 8	CFs: 35% Control: 15%	6 weeks	Chocolate bar and cocoa beverage; *Total flavs: 444mg* *Epicatechin: 107mg*	Matched placebo bar and beverage *Total flavs: 19.6mg* *Epicatechin: 4.7mg*	Skeletal muscle (forearm)
(q) Muniyappa 2008 [69]	Crossover (1 week)	Mild-moderate hypertensives	*n* = 20 (13 WE, 7 BA)	51± 6.71	60%	2 weeks	2*31 g cocoa powder with 150 ml water *Total flavs: 902mg* *Epicatechin: 174mg*	2*31 g matched placebo powder with 150 ml water *Total flavs: 28mg* *Daily epicatechin: 2mg*	Skeletal muscle (forearm)
(o) Decroix 2018b [66]	Crossover (1 week)	Trained cyclists	*n* = 14	30.7 ± 3.1	0%	1 week	4 capsules *Total flavs: 1765mg* *Epicatechin: 100mg*	4 matched capsules *Total flavs: 0mg*	Skeletal muscle (leg)/ cerebral
(r) Shaw 2020 [70]	Crossover (2 weeks)	Trained cyclists	*n* = 12	35 ± 12	16.7%	2 weeks	120 g (60 g*2) per day 72% dark choc *Total flavs: 1788mg* *Epicatechin: 37.4mg*	120 g (60 g*2) per day non-choc placebo *Total flavs: 0mg*	Skeletal muscle (leg)/ cerebral
(s) Sumiyoshi 2020 [71]	Parallel	Healthy	CFs: *n* = 10 Control: *n* = 8	20–31	27.8%	30 days	24 g 70% dark chocolate *Total flavs: 540mg* *Epicatechin: 34.8mg*	24.5 g white chocolate *Total flavs: ND*	Cerebral

*Data shown are mean ± SD. CAD = coronary artery disease*,* WE = White European*,* BA = Black African. Flavs = flavanols*

### Acute studies

Studies using a single dose of CFs tested effects across the cutaneous (*n* = 5 [45, 56, 61, 62, 72]), skeletal muscle (*n* = 3 [55, 63, 72]), retinal (*n* = 2 [64, 65]), and cerebral microcirculations (*n* = 4 [57–60]), and their characteristics are shown in Table 2. Of the 13 studies, 12 had a cross-over design (with wash-out periods ranging from 72 h to 2 weeks), whilst one was run with parallel groups [72]. Sample sizes of the included studies ranged between 7 and 22 individuals per group. The majority of studies were conducted in healthy populations (age range: 18–65 years), with many focussed on young adults (< 30 years), whilst others included clinical populations, such as type 2 diabetes [56] or peripheral artery disease (PAD) [61]. One study compared between ethnic groups with differing CVD risk [62], and only one other study reported the ethnicity of participants [55]. Most studies included males and females [56, 57, 61–65]; 4 studies included only males [55, 58, 60, 72] and just one study was on females only [45].

The source of CFs in these acute studies was dark chocolate (*n* = 3 [61, 64, 65]), capsules (*n* = 4 [56, 57, 59, 63]), or a cocoa beverage (*n* = 6 [45, 55, 57, 58, 61, 62, 64, 65, 72]), with total flavanol doses ranging from 80 to 1350 mg. (-)-Epicatechin dosage was also reported in 11 studies, with this ranging from 45 to 447 mg. Acute effects were assessed within 60–120 min in all studies, aligning with the peak CF concentration in the blood at this time [44, 45], with some also following effects for up to 6 h post-intervention. Whereas most studies asked participants to fast for a minimum of 8 h prior to intervention, 4 studies administered the CFs alongside a meal or carbohydrate-rich drink [56–59], which they reported to increase CF absorption [46]. 8 studies compared effects of CF on resting blood flow, whilst others looked at the effects of CFs on microvascular vasodilator responses to stimuli such as RH (*n* = 3 [56, 61, 72]), mental stress (*n* = 3 [55, 58, 59]), local heating (*n* = 1 [62]), exercise (*n* = 1 [58]), hypercapnia (*n* = 1 [60]), or hypoxia (*n* = 1 [57]).

### Chronic studies

Seven studies used a longer-term CF intervention and compared effects across a range of 7 days to 12 weeks; their characteristics are shown in Table 3. Similar to the acute studies they incorporated a range of vascular beds from cutaneous (*n* = 2 [68, 72]), skeletal muscle (*n* = 5 [66, 67, 69, 70, 72]), and cerebral microcirculation (*n* = 3 [66, 68, 70–72]). 3 of the 7 studies used a cross-over design, with wash-out periods of 1–2 weeks, and the remaining 4 studies used parallel groups. Sample sizes ranged from 10 to 20 per group. Healthy individuals made up the majority of volunteers, with some focussing on trained individuals and others including the effects within coronary artery disease patients [67] and hypertensives [69]. Studies were conducted mainly in mixed sex (*n* = 4) groups, with two on males only [66, 72] and one on females only [68]. Only one study reported the ethnicities of participants: a mixture of White Europeans and Black Africans [69].

Daily CF doses were obtained from dark chocolate (*n* = 2 [70, 71]), cocoa beverages (*n* = 3 [68, 69, 72]), capsules (*n* = 1 [66, 70, 71]), or a combination of chocolate and beverages (*n* = 1 [67]). Total daily flavanol intake ranged from 329 to 1788 mg and daily (-)-epicatechin dosage was 35–174 mg. Studies compared effects of CFs on resting values (*n* = 4 [67–69, 72]), or after vasodilator stimuli such as RH (*n* = 2 [67, 72]), exercise (*n* = 3 [66–70, 72]), acetylcholine infusion (*n* = 1 [67]), insulin infusion (*n* = 1 [69]), or mental stress (*n* = 1 [71]).

### Study findings

#### Acute CF interventions

**Fig. 3:**
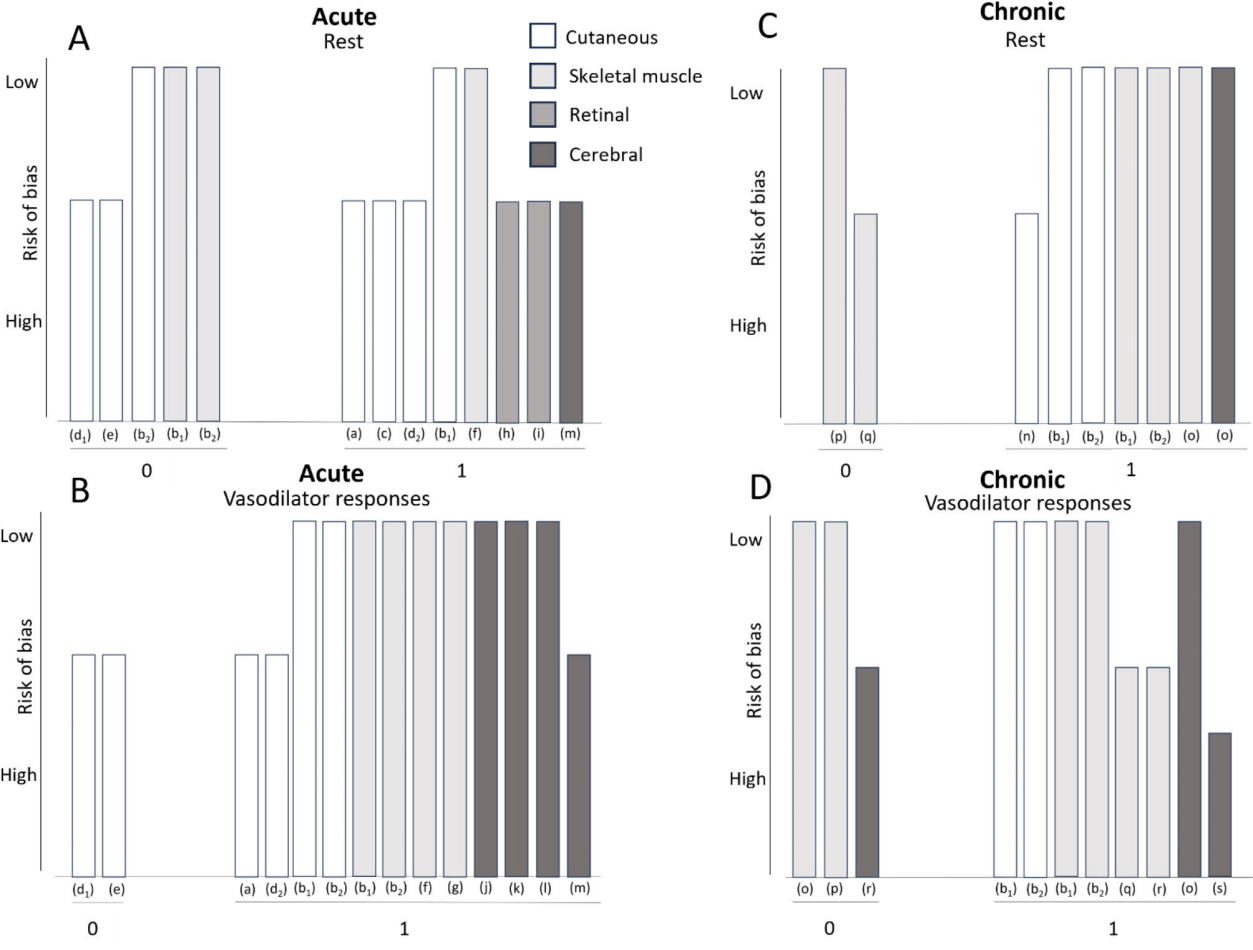
Harvest plots presenting direction of effect for subgroups of all studies, showing acute effects **(A)** at rest and **(B)** on vasodilator responses, and chronic effects **(C)** at rest, and **(D)** on vasodilator responses. The colours of the bars represent the microvascular bed studied. Tallest bars= ‘low risk of bias’, shortest bars= ‘high risk of bias’. ‘1’ represents a positive effect of cocoa flavanols (CFs), and ‘0’ represents increased outcomes with placebo vs. CFs

Table 4 summarises the findings in relation to key outcome measures for all acute studies, grouped according to the vascular bed investigated and risk of bias. Analysis by vote counting using subgroups from a Harvest plot (Fig. 3) suggests there is evidence of a beneficial effect of CFs on vasodilator responses, with 12 out of 14 subgroups showing enhanced vasodilator responses following CF intervention (85.7% (80.4–91.0%), *p* = 0.013). Importantly, all of the 9 groups which were ‘low risk of bias’ showed a positive effect of CFs on vasodilator responses (100%, p = 0.004). By contrast, the effect at rest was less pronounced with benefits shown in 8 out of 13 subgroups (61.5% (52.2–70.9%), p = 0.581), an effect which was not statistically significant. A total of 11 acute studies included effects in healthy, young populations: a beneficial effect of CFs was shown at rest in 6 out of 9 subgroups, but this was not statistically significant (66.7% (54.1–79.2%), p = 0.508), whilst an improvement in vasodilator responses was evident in 9 out of 11 subgroups (81.8% (74.2–89.4%), p = 0.065). Indeed, considering only studies with ‘low risk of bias’, all 8 subgroups showed improvement in vasodilator responses with CFs (100%, p = 0.008). Further statistical analysis within each vascular bed studied, or within the subgroups with increased CVD risk was not possible due to the small number of comparable studies.

Within the cutaneous microcirculation, three studies found no significant effect of CFs on resting blood flow [61, 62, 72], whereas Neukam et al. showed a 1.7-fold increase in resting cutaneous blood flow in the forearm of healthy females [45] and Bapir et al. found that CFs increased baseline microvascular diameter in the skin of the feet but not hands [56]. The latter study was conducted in healthy and type-2 diabetes patients; diabetic patients showed blunted peak RH relative to healthy individuals, but there was no effect of CFs on peak RH in the hands or feet of the combined population [56]. Similarly, smaller cutaneous vasodilator responses were demonstrated in elderly compared to young males, but peak RH was increased by CFs in both groups [72]. By contrast, no effect of CFs was found on peak cutaneous RH in PAD patients [61].

Furthermore, smaller peak FBF measured by VOP was also observed in elderly versus young males, with peak RH being increased by CFs in both groups [72]. Whilst VOP provides a measure of whole limb blood flow, muscle resistance vessels are thought to contribute most to this response [73]. Furthermore, there was evidence of increased FBF measured by VOP at rest and during mental stress in a group of young males [55]. On the other hand, Santos et al. found no significant effect of CFs on reperfusion rate or peak SO_2_ measured by NIRS, following a post-exercise vascular occlusion test [63].

Within the retinal microvasculature, both studies showed no effect of dark, compared to white chocolate, in young healthy populations of mixed sex [64, 65]. Finally, in the cerebral cortical microvasculature, there was evidence using NIRS of greater increases in oxyHb following CF supplementation in response to a cognitive task, as well as to hypoxia and hypercapnia, but no significant effect during exercise, in young mixed or only male populations [57, 58, 60].

**Table 4:** Findings from acute studies, including reported outcome measures for placebo and cocoa interventions and the direction of effect

	Monitoring technique/ outcome measure	Rest/ vasodilator stimulus	Outcome measures (placebo)	Outcome measures (cocoa flavanols)	*P* values	Vote counting (direction of effect)	Risk of bias
Cutaneous							
(b) Heiss 2015 [72]	Laser doppler perfusion imaging (LDPI)	Rest	(b1) 42 ± 4.69/42.1 ± 4.69 PU (b2) 41 ± 4.47/44 ± 0.44 PU	(b1) 38 ± 4.69/40 ± 4.69 PU (b2) 38 ± 4.47/39 ± 4.47 PU		1 0	Low risk
		RH peak	(b1) 259 ± 70.4/270 ± 79.7 PU (b2) 186 ± 35.8/186 ± 35.8 PU	(b1) 257 ± 65.7/292 ± 70.4 PU (b2) 184 ± 67.1/200 ± 62.6 PU	*p* < 0.05 *p* < 0.05	1 1	
(c) Neukam 2007 [45]	Laser Doppler (cutaneous blood flow)	Rest	*22 ± 15/22 ± 10 AU*	*30 ± 10/50 ± 8 AU*	*p* < 0.05	1	Some concerns
(a) Bapir 2022 [56]	OCT (hands/ feet, mean vessel diameters)	Resting	Foot: 46 ± 4/43 ± 4 μm Hand: 57 ± 4/49 ± 9 μm	Foot: 44 ± 4/44 ± 4 μm Hand: 48 ± 4/48 ± 4 μm	*p* < 0.001 *p* = 0.371	1 1	Some concerns
		RH peak	Foot: 49 ± 4/47 ± 4 μm Hand: 57 ± 4/55 ± 4 μm	Foot: 51 ± 9/50 ± 4 μm Hand: 57 ± 4/57 ± 4 μm	*p* = 0.751 *p* = 0.120	1 1	
(d) Kim 2020 [62]	Laser Doppler (forearm)	Rest (%CVC max)	(d_1_) 11.15 ± 1.44 (d_2_) 8.16 ± 2.56	(d_1_) 9.73 ± 1.3 (d_2_) 9.99 ± 2.31		0 1	Some concerns
		Local heating (flux/mmHg)	(d_1_) 3.21 ± 0.43 (d_2_) 2.85 ± 0.17	(d_1_) 3.03 ± 0.25 (d_2_) 3.04 ± 0.26	*p* = 0.4 *p* < 0.01	0 1	
(e) Hammer 2015 [61]	Laser Doppler (forearm) Median (IQR)	Rest	0.22(0.13–0.47)/0.41(0.24–0.51) AU	0.32(0.18–0.60)/0.31(0.25–0.55) AU	*p* = 0.78	0	Some concerns
		RH peak	0.89(0.58–1.49)/1.24(0.85–1.79) AU	1.22(0.84–1.87)/1.18(0.7–2.27) AU	*p* = 0.69	0	
Skeletal							
(f) Baynham 2021 [55]	VOP (forearm, % change pre-post)	Rest	-0.38 ± 0.28%	0.34 ± 0.83%	*p* < 0.001	1	Low risk
		Stress	0.26 ± 0.66%	1.46 ± 1.81%	*P* = 0.002	1	
(b) Heiss 2015 [72]	VOP (forearm)	Rest	(b_1_) 1.5 ± 0.47/1.5 ± 0.47 (b_2_) 1.1 ± 0.45/0.9 ± 0.45	(b_1_) 1.7 ± 0.47/1.6 ± 0.47 (b_2_) 1.5 ± 0.45/1.2 ± 0.45		0 0	Low risk
		RH peak	(b_1_) 13.7 ± 7.50/14.3 ± 8.44 (b_2_) 11.3 ± 5.36/10.8 ± 6.71 (all ml/100 ml*min)	(b_1_) 13.2 ± 2.81/16.2 ± 4.69 (b_2_) 10.9 ± 5.36/12.3 ± 6.71 (all ml/100 ml*min)	*p* < 0.05 *p* < 0.05	1 1	
(g) Santos 2023 [63]	NIRS-derived SO_2_ (forearm)	Vascular occlusion test post-exercise	Reperfusion rate (%/s): 2.03 ± 0.46/ 2.02 ± 0.59	Reperfusion rate (%/s): 2.14 ± 0.58/ 2.35 ± 0.92	*p* = 0.488	1	Low risk
Retinal							
(h) Scuderi 2020 [64]	OCT-A (change from baseline @2hrs, SCP whole density)	Rest	0.586 ± 2.67%	0.739 ± 1.816%	*p* = 0.317	1	Some concerns
(i) Siedlecki 2019 [65]	OCT-A (superficial retinal plexus)	Rest (vessel density)	47.5 ± 2.6%	48.0 ± 2.7%	*p* = 0.56	1	Some concerns
Cerebral							
(j) Decroix 2018a [59]	fNIRS (oxyHb, right PFC)	Stroop task	*1.2 ± 1.5 AU*	*1 ± 1.5 AU*		0	Low risk
(k) Decroix 2016 [58]	fNIRS (oxyHb)	Cognitive task	*1.5 ± 0.3 AU*	*2 ± 0.3 AU*	*p* = 0.02	1	Low risk
		Exercise	*12 ± 1.5 AU*	*12.5 ± 1.5 AU*		1	
(l) Gratton 2020 [60]	fNIRS (change in oxyHb, at 3–4)	Hypercapnia	*18 ± 41.2/11 ± 24.7 AU*	*17 ± 33.0/36 ± 33.0 AU*	*p* = 0.03	1	Low risk
(m) Bloomfield 2023 [57]	fNIRS (oxyHb, normalised to normoxic baseline)	Rest	0.8 ± 1.0 µmol	1.7 ± 2.3 µmol	*p* = 0.005	1	Some concerns
		Hypoxia	-4.0 ± 3.6 µmol	-1.3 ± 2.7 µmol		1	

Table is ordered by risk of bias within sub-sections for each vascular bed. Outcome measures are shown as mean ± SD for pre/post intervention unless otherwise stated, and data shown in italics has been estimated from graphs. OCT-A = optical coherence tomography angiography. CVC = cutaneous vascular conductance. IQR = interquartile range. VOP = venous occlusion plethysmograph. (f)NIRS= (functional) near-infrared spectroscopy

### Chronic CF interventions

Table 5 summarises the direction of effects for all chronic studies. Overall, by vote counting subgroups (Fig. 3), 7 out of 9 subgroups favoured a beneficial CF effect at rest (77.7% (67.5–88.0%), *p* = 0.180) and 8 out of 11 showed a beneficial effect on vasodilator responses (72.7% (63.4–82.0%), *p* = 0.227), but these did not reach statistical significance. Considering only subgroups from studies with ‘low risk of bias’, 6 out of 7 showed a beneficial effect at rest (85.7% (75.1–96.3%), *p* = 0.125), and 5 out of 7 showed enhanced vasodilator responses following CF supplementation (71.4% (56.5–86.4%), *p* = 0.453), but these are also not statistically significant. There were 5 chronic studies which included young, healthy populations; of these, 7 subgroups tested effects of CF supplementation at rest and all showed improvements (100%, *p* = 0.016), which were statistically significant. 7 out of 9 subgroups testing vasodilator responses showed improvement with chronic CF supplementation (77.8% (71.2–84.4%), *p* = 0.180), but no statistical significance was reached. Considering only the 6 subgroups from studies with ‘low risk of bias’, there was evidence of microvascular improvements at rest in all 6 (100%, *p* = 0.031), and 5 out of 6 showed improved vasodilator responses (83.3% (77.6–89.0%), *p* = 0.219), but this effect was not statistically significant. The remaining studies included populations with increased risk of CVD, but the number of subgroups was too limited to conduct statistical analysis. Additionally, studies investigating effects on at-risk populations focused on different microvascular beds, limiting any further meaningful analysis.

Studies of the cutaneous microvasculature show mixed findings for the effects of chronic CF on resting blood flow; Heinrich et al. found increases at rest in the forearm cutaneous circulation of healthy women after 6 and 12 weeks of supplementation [68], whereas Heiss et al. showed no difference at rest in young or elderly males after 14 days [72]. The latter study also found no difference in resting FBF in forearm muscle as measured by VOP, although peak cutaneous and whole-limb RH was enhanced by CFs as measured by Laser Doppler and VOP respectively [72]. In contrast, no effect of 6 weeks CF supplementation on FBF measured by VOP was found at rest or at peak RH or exercise in CAD patients [67]; indeed, responses to acetylcholine infusion were smaller following CFs versus control in this group [67]. Furthermore, in a group of hypertensive patients of mixed sex and ethnicity there was no effect of CFs after 2 weeks at rest or following insulin infusion on capillary blood flow [69].

Two studies investigated the effects of CFs on leg muscle and cerebral oxygenation measured by NIRS during exercise in trained cyclists: Shaw et al. found significant increases in leg, but not cerebral oxyHb, during exercise in a hypoxic chamber after two week’s CF supplementation [70], whereas Decroix et al. found that one week of CFs increased SO_2_ response to hypoxia and exercise in the cerebral microvascular but not leg muscle, despite increases in resting SO_2_ in both tissues [66]. Finally, Sumiyoshi found no significant effect of 30 days CFs on cerebral oxyHb during cognitive tasks [71], although this result should be viewed with caution given the study was judged ‘high risk of bias’.

**Table 5:** Findings from chronic studies, including reported outcome measures for placebo and cocoa interventions and the direction of effect

	Monitoring technique/ outcome measure (units)	Rest/ vasodilator stimulus	Outcome measures (control) Mean ± SD pre/post, unless otherwise stated	Outcome measures (cocoa flavanols) Mean ± SD pre/post, unless otherwise stated	*P* values	Vote counting (direction of effect)	Risk of bias
Cutaneous							
(b) Heiss 2015 [72]	Laser doppler perfusion imaging (LDPI, forearm)	Rest	(b_1_) 42 ± 4.7/39 ± 4.7 PU (b_2_) 39 ± 4.5/40 ± 4.5 PU	(b_1_) 39 ± 4.7/40 ± 4.7 PU (b_2_) 40 ± 4.5/39 ± 4.5 PU		1 1	Low risk
		RH	(b_1_) 260 ± 103/258 ± 79.7 PU (b_2_) 178 ± 22.4/180 ± 22.4 PU	(b_1_) 307 ± 65.7/ 296 ± 79.7 PU (b_2_) 214 ± 53.6/213 ± 53.6 PU	*p* < 0.05 *p* < 0.05	1 1	
(n) Heinrich 2006 [68]	Laser Doppler (blood flow)	Rest	6 weeks: 17 ± 9/17 ± 6 AU 12 weeks: 17 ± 9/16 ± 6 AU	6 weeks:16 ± 7/24 ± 12 AU 12 weeks: 16 ± 7/32 ± 16 AU	*p* < 0.05 *p* < 0.05	1 1	Some concerns
Skeletal muscle							
(b) Heiss 2015 [72]	VOP (forearm)	Rest	(b_1_) 1.5 ± 0.47/1.4 ± 0.47 (b_2_) 0.8 ± 0.045/0.9 ± 0.045	(b_1_) 1.6 ± 0.47/1.6 ± 0.47 (b_2_) 1.1 ± 0.45/1.1 ± 0.045		1 1	Low risk
		RH	(b_1_) 13.7 ± 7.97/13.8 ± 7.04 (b_2_) 11.6 ± 7.60/11 ± 6.71 (All ml/100 ml*min)	(b_1_) 16.9 ± 2.81/16.9 ± 5.16 (b_2_) 14.0 ± 6.26/13.9 ± 4.92 (All ml/100 ml*min)	*p* < 0.05 *p* < 0.05	1 1	
(o) Decroix 2018b [66]	NIRS (SO_2_)	Rest	*59.2 ± 2%*	*60.2 ± 2%*		1	Low risk
		Hypoxia	*60.5 ± 1.5%*	*58.5 ± 1%*		0	
		Exercise	*57 ± 1.5%*	*56.8 ± 1.5%*		0	
(p) Farouque 2006 [67]	VOP (forearm)	Rest	2.49 ± 1.12/2.8 ± 1.57	2.82 ± 01.57/2.47 ± 0.98		0	Low risk
		ACh infusion	*5 ± 0.2/8 ± 1*	*8 ± 1/8.2 ± 1.5*	*p* < 0.05	0	
		RH	28.09 ± 8.23/28.05 ± 7.83	31.15 ± 1.62/30.41 ± 7.38		0	
		Exercise	24.08 ± 9.48/23.06 ± 8.18 (all ml/100 ml/min)	22.72 ± 2.1/24.88 ± 9.84 (all ml/100 ml/min)		0	
(q) Muniyappa 2008 [69]	Doppler (capillary blood flow)	Rest	0.68 ± 1.03 AU	0.51 ± 0.31 AU		0	Some concerns
		Insulin infusion	0.67 ± 0.49 AU	0.74 ± 0.40 AU	*p* = 0.31	1	
(r) Shaw 2020 [70]	NIRS (average oxyHb, change from baseline)	Exercise at simulated altitude	Sub max: -5.67 ± 5.34 AU Time trial: -5.50 ± 5.31 AU	Sub max: -0.84 ± 3.56 AU Time trial: -1.02 ± 3.41 AU		1 1	Some concerns
Cerebral							
(o) Decroix 2018b [66]	NIRS (SO_2_)	Rest	*61.5 ± 1%*	64 *± 1%*	*p* < 0.05	1	Low risk
		Hypoxia	*59 ± 1%*	*61.5 ± 1%*	*p* < 0.05	1	
		Exercise	*62 ± 0.5%*	*64 ± 1%*	*p* = 0.004	1	
(r) Shaw 2020 [70]	NIRS (average oxyHb- change from baseline)	Exercise at simulated altitude	Sub max: 10.7 ± 5.06 AU Time trial: 3.99 ± 8.34 AU	Sub max: 24.4 ± 20.9 AU Time trial: 10.8 ± 17.6 AU		0	Some concerns
(s) Sumiyoshi 2020 [71]	NIRS (oxyHb, at end of word tests)	Cognitive tasks	*45 ± 15/40 ± 15 AU*	*60 ± 10/45 ± 5 AU*		1	High risk

Table is ordered by risk of bias within sub-sections for each vascular bed. Outcome measures are shown as mean ± SD for pre/post intervention unless otherwise stated, and data shown in italics has been estimated from graphs. VOP = venous occlusion plethysmography. NIRS = near-infrared spectroscopy

## Discussion

This systematic review explored for the first time the role of CFs in regulating microvascular function in humans, taking into consideration findings at rest and in response to vasodilator stimuli across a range of microvascular beds (cutaneous, muscle, brain, retinal). Overall, the relatively small number of studies with a focus on microvasculature revealed heterogeneous results and prevented meta-analysis from being conducted according to age range or health status. Despite these limitations, there is evidence to suggest that CFs may improve microvascular function, particularly in young, healthy individuals, which was the population sampled in the majority of included studies. Specifically, there were statistically significant benefits of acute CFs intake on vasodilator responses, but not at rest, in young healthy populations. Importantly, this effect was replicated when only studies with low-risk bias were considered. The benefits within the microvasculature seem to be greater following an acute dose of CFs rather than supplementation over a period of days or weeks. In contrast with acute interventions, for chronic studies a statistically significant benefit was apparent only at rest, and not in response to vasodilator stimuli, in young, healthy groups. The lack of data in at-risk populations makes it difficult to draw conclusions within this group compared to healthy individuals, and the heterogeneity of vascular beds studied means that no firm conclusions can be reached within specific microvascular beds.

Although some studies included effects at rest, as well as on vasodilator responses, many did not account for differences in the resting or baseline value when considering the peak vasodilator responses. Therefore, it is difficult to establish whether the magnitude of dilatation was indeed increased, or whether this was confounded by the effect at baseline; theoretically if the size of the effect at rest was of similar magnitude to that on the dilator response, this may indicate that there was no additional benefit of CFs in inducing vasodilation in response to the stimulus, beyond its effect at baseline. Importantly, all acute studies which showed a positive effect of CFs at rest also found an increase in peak vasodilator responses [55–57, 62], with others showing no benefit of CFs at rest or on vasodilator responses [61, 62], and only one study showing that CFs increase FBF at peak RH but not at rest [72]. Despite this, vote counting analysis indicated a significant effect of CFs on vasodilator responses but not at rest, suggesting that functionally, the increase in blood flow evoked by the various stimuli was enhanced by CFs.

Nonetheless, there is evidence of an effect of CFs at rest in some studies included within the present review, suggesting that CFs may attenuate mechanisms that induce vasoconstrictor tone at rest and/or facilitate those that induce tonic vasodilation. Since NO exerts a tonic dilator influence at rest, as demonstrated from the local effects of NOS inhibition [74], the dilator influence of CFs on the resistance vessels at rest may be due to facilitation of the tonic release or actions of NO directly [11, 75, 76], or indirectly by inhibiting the release and action of ET [77]. There is also some evidence to suggest flavanols may stimulate the cyclooxygenase pathway, leading to increased production of prostacyclin [78], which also promotes vasodilation, or alternatively by influencing (endothelium-derived hyperpolarising factors (EDHFs) or by acting as antioxidants [79]. Thus, it is possible that CFs may also facilitate their tonic effects via these mechanisms.

Considering studies investigating the effect of CFs within the macrovasculature, improvements in dilator responses after CF intake have been widely documented [23, 25], as evidenced by consistent increases in brachial FMD [22, 23], despite no evidence of an effect on resting vascular tone in these studies. Since FMD is thought to be largely NO-mediated, though not entirely (the vasodilator effect is not completely blocked by NOS inhibition [80]), this suggests that CFs act, at least partly, by increasing NO bioavailability. Although the exact mechanisms are not well established, there is evidence to suggest calcium-mediated activation of signalling pathways within the endothelium leading to activation of endothelial nitric oxide synthase [81, 82]. Furthermore, CFs are proposed to act by modulating antioxidant pathways, reducing levels of circulating reactive oxygen species, thereby increasing NO bioavailability [3, 79].

In relation to the present review, some of the included studies found that CFs augmented RH within the cutaneous or skeletal muscle microvasculature [56, 63, 72], whereas others showed no effect [61, 67]. Since the contribution of NO to peak and RH in the forearm and cutaneous microvasculature is limited [83], this raises the possibility that CFs may augment RH by facilitating other dilator mechanisms that contribute to peak RH including prostaglandins and EDHFs [83]. Given that there are known interactions between dilator pathways, it is challenging to firmly establish the underpinning mechanisms of action of CFs within the microvasculature [84, 85]. Similarly, some of the included studies showed a significant effect of CFs on limb vasodilator response to exercise [70] but not others [66, 67]. Again the contribution of NO to the muscle vasodilator response to exercise is thought to be minor, since previous studies using NOS inhibition have shown little effect on peak exercise hyperaemia [86]. Rather, prostaglandins and EDHFs are proposed to also play a role in exercise hyperaemia and it is recognised there is redundancy between the various mediators [87, 88]. Thus, the reported findings on EH in microcirculation may be explained by CFs influencing any of these vasodilator mechanisms.

Interestingly, NO has been shown to contribute to limb vasodilator responses to mental stress [89–91], with NOS inhibition partially blocking forearm muscle vasodilation in response to mental stress [92]. Thus, in the context of findings in the present review, where a beneficial effect of CFs on muscle vasodilator responses to mental stress was identified [55], it supports the idea that CFs can act via NO within the microvasculature but raises the possibility of modulation of other NO-independent vasodilator mechanisms.

Despite evidence that changes in vascular function are closely related to blood pressure [93], many studies included in the present review did not consider changes in blood pressure [45, 57, 58, 68]. Only one study which found a significant effect of CFs on microvascular function also demonstrated a significant decrease in diastolic blood pressure [72], whilst most other studies which reported a significant effect of CFs on vascular function found no change in blood pressure [55, 60, 66, 67].

In the cerebral microcirculation, a role of NO alongside with other vasodilator factors has been postulated in responses to hypoxia and hypercapnia [94, 95]. The present review showed that CFs augmented cerebral vasodilation under these conditions [57, 60, 66], whereas findings in relation to exercise and cognitive tasks were more varied [58, 59, 66, 70, 71], likely due to the more complex mechanisms behind these responses. Altogether, the fact that microvascular effects seem less clear than previous evidence in studies of the macrovasculature suggests that different mechanisms may be at play. Further studies are required to establish the mechanisms by which CFs may facilitate cerebral vasodilation at the microvascular level within healthy, young individuals.

It has been suggested that in individuals with CVD (or with higher CVD risk), the higher prevalence of free radicals and oxidative stress may make this population more susceptible to the benefits of CF supplementation [3]. For example, systematic reviews have demonstrated greater blood-pressure lowering effects of CFs in hypertensives compared to normotensives [7]. Comparatively, Woodward et al. suggested that benefits of CFs in the microvasculature were greater in healthy individuals than those with high CVD-risk, since effects observed in healthy individuals were not replicated in patient populations in some studies included within their review [96]. Within the current review, this idea is supported by a study, in which responses to cutaneous local heating following CFs intake were increased only in Black Africans [62], known to be at increased CVD risk compared to White Europeans [97, 98].On the other hand, other studies report no differences in the effects of CFs on RH evoked in the cutaneous or skeletal muscle between healthy young populations, and type-2 diabetics [56] or elderly individuals [72]. These apparent disparities between individual studies must be treated with some caution, particularly given the small sample sizes and potential concerns of bias. It would therefore be beneficial to conduct future randomised controlled human trials directly comparing the role of CFs within the microvasculature in populations of differing CVD risk, in order to determine individuals for whom CF supplementation would be most beneficial.

In regard to the length of CF supplementation, it appears that CFs are more efficacious in modifying microvascular responses in the hours immediately following an acute dose, rather than after prolonged supplementation over a period of days or weeks. Even though enhanced vasodilator responses were reported after chronic CF supplementation in 8 subgroups [66, 69, 70, 72], these effects did not reach statistical significance in analysis by vote counting within the present review, probably reflecting the small number of studies and the fact that some studies reported no effect of chronic CF supplementation. This contrasts to previous findings for brachial FMD, which is well-established to be modified by CFs both acutely and chronically across many different studies [21, 22] ranging from seven days to six weeks duration. Thus, the findings of the present review raise the possibility that the microvasculature may be less susceptible to long-lasting effects of CFs than conduit arteries. Indeed, it appears that the benefits of CFs within the microvasculature are short-lasting and coincide with the peak increases in flavanol metabolites and plasma nitroso species - recognised markers of NO metabolism, 1–3 h after supplementation [11]. Since plasma flavanols were not tested in the majority of chronic studies, the actual circulating concentrations are unclear, which may partly explain the limited effects observed compared to acute studies. Nonetheless, there is evidence that high levels of habitual CF intake result in maintained elevation of urinary flavanol metabolites consistent with the intake quantities [11]. It is therefore possible that a higher dose of CFs is required to exert effects within the microvasculature compared to the doses previously reported in FMD studies.

The lack of clear effects in chronic studies may also be attributed to the variability in dilator responses measured over time, which makes it difficult to detect changes outside of individual fluctuations. This issue is likely to be accentuated by the small sample populations included in each study, because they are less well powered to detect small effects. A further consideration for chronic studies is that it is difficult to control participants’ overall diet during a longer time-period as they are likely to continue to consume flavonoids from other sources, such as fruit and vegetables. For this reason, chronic studies should take the background diet into account since the influence of supplementary CFs may be greater in those with otherwise low dietary flavonoid intake, as shown for their effects on memory improvement [99]. Nonetheless, these issues also apply to chronic FMD studies in which beneficial effects of CFs are still detectable; this further supports the idea that CFs may exert effects on the conduit vessels that are greater than those on the microvasculature. It is important to note that the doses used in the studies included in this review are much higher than estimated typical dietary intakes [100]. Thus, in order to confer such effects as those reported, intake levels would need to be increased at least to the recommended daily level of 400-600 mg/day shown to improve cardiometabolic health. This could be attained from a mixture of flavanol-rich sources including tea, cocoa, apples and blackberries in order to maximise benefits [24]. Importantly, there are no known adverse effects of flavanol consumption with previous evidence showing that consumption of up to 2000 mg/day is well tolerated in healthy adults [101].

### Limitations

The conclusions of the present systematic review are limited due to the relatively small number of studies available and their heterogeneity. There was not sufficient data for any single measure of microvascular function to allow for a meta-analysis to be performed, which would have provided a better, more reliable analysis of the efficacy of CFs on human microvasculature. Instead, the limited available data from all studies led us to conduct analysis by vote-counting, as an alternative to more quantitative meta-analyses [51]. Unfortunately, this is the lowest recommended level of synthesis, and provides no information on the magnitude of effect. Indeed, when following this method all studies, even those where there was no significant effect had to be categorised, resulting in inclusion of data where minimal changes were observed. Furthermore, vote-counting does not account for relative differences in effect sizes between studies and is less powerful than other methods, which are used to combine p values [102]. As such, by using vote-counting, we were only able to answer whether there is evidence of an effect, rather than provide information on the magnitude of the effect.

There were also limitations within the design of the studies included in the present analysis, which further complicates the interpretation of their findings. Firstly, many studies reported findings for mixed populations without incorporating any sex comparisons, despite evidence that vasodilator responses differ between males and females [103–105]. Furthermore, it is well established that some ethnic groups are at higher risk of CVD (such as South Asians and Black Africans) and exhibit blunted vasodilator responses [97, 106, 107], but most studies did not report the ethnicity of the participants. Moreover, there is evidence that differences in the effect of CFs can be detected between different ethnicities, particularly White Europeans and Black Africans [62]. Advancing age is another common CVD risk factor that is often overlooked, with some studies reporting data from subjects from a very wide age range, despite extensive evidence of impaired microvascular vasodilator responses in elderly, compared to young populations [72, 108]. As such, the variability of individual responses within a small sample group of mixed sex, ethnicity or age may mask any potential effect of CFs within the population.

### Future directions

A key issue which should be addressed is the heterogeneity of study populations. Future studies of CFs in the microvasculature should stratify by sex, age, ethnicity, and health status in order to gain more valuable insight within targeted populations, without confounding the effects of variable baseline vascular health. Future studies should also assess the habitual diet of participants, and take into account background flavonoid intake, which may influence the magnitude of effects induced by supplementary CFs on microvasculature, as has been demonstrated for hippocampal-dependent memory [99]. It would be of interest to determine whether there is a correlation between habitual flavanol intake and microvascular effects of CFs, in order to identify the individuals who may benefit most from CF supplementation.

Future research should also establish the optimal dose for beneficial effects of CF on the microcirculation, by conducting more controlled dose-effect intervention studies. For example, there is evidence of a dose-dependent response relationship between CFs intake and beneficial effects on FMD and on blood pressure [25]. This seems likely to be the case for microcirculatory function, but the minimum efficacious dose might be different for macro and microvasculature. This might also be particularly relevant for chronic studies, where the effects of CF on microvascular responses are less pronounced. In order to address this issue, long-term supplementation using a range of doses, with assessment of vascular function and monitoring of circulating flavanols at regular intervals would provide insight into the level of CFs required to exert significant improvements. Such information would help inform how the intake of flavanols might be translated into daily consumption levels and may guide future dietary recommendations.

Finally, it will be of key importance to establish the mechanisms of action underpinning the effects of CFs within the microvasculature and how this relates to the more established effects in larger vessels. A role for NO in the vascular effects of CFs on brachial artery FMD has been postulated [11, 75], but the complexity of the vasodilator factors involved in microvascular responses to RH, exercise, hypoxia and mental stress where the effects of CFs are less consistently observed and where the contributions of NO are not so prominent, raises the possibility that CFs may also influence other dilator factors, directly or due to their interplay with NO [84, 109]. In that regard, it would be useful to establish whether CFs are still effective within the microvasculature when NO activity is inhibited both in isolation and alongside inhibition of other pathways (such as prostaglandin synthesis inhibition by cyclooxygenase inhibitors), in order to elucidate whether CFs are acting via other pathways in this instance or whether effects are still NO-dependent.

## Conclusion

Overall, there is evidence to suggest that CFs may reduce resting microvascular tone and improve vasodilator responses across skeletal muscle, skin and cerebral circulation, particularly when administered acutely. No major differences were detected in efficacy of CFs between healthy and at-risk populations, but there is clearly a need to conduct studies that formally compare these. Effects were detected in microvascular responses that are not solely mediated by NO, implying that not all the effects of CFs are due to direct action via NO, though they may still be NO-dependent due to the interaction of vasodilator pathways.

## Electronic supplementary material

Below is the link to the electronic supplementary material.

Supplementary Material 1

## Data Availability

No datasets were generated or analysed during the current study.

## Abbreviations

BA: Black African
CAD: Coronary artery disease
CF: Cocoa flavanol
CVC: Cutaneous vascular conductance
CVD: Cardiovascular disease
EDD: Endothelium-dependent dilatation
EDHF: Endothelium-derived hyperpolarising factor
FMD: Flow-mediated dilatation
Hb: Haemoglobin
IQR: Interquartile range
NIRS: Near-infrared spectroscopy
NO: Nitric oxide
OCT-A: Optical coherence tomography angiography
PAD: Peripheral artery disease
RCT: Randomised controlled trial
RH: Reactive hyperaemia
SD: Standard deviation
SO_2_: Oxygen saturation
T2DM: Type-2 diabetes mellitus
VOP: Venous occlusion plethysmography
WE: White European
